# Deoxynivalenol-Induced Proinflammatory Gene Expression: Mechanisms and Pathological Sequelae

**DOI:** 10.3390/toxins2061300

**Published:** 2010-06-01

**Authors:** James J. Pestka

**Affiliations:** 1 Department of Microbiology and Molecular Genetics, Michigan State University, East Lansing, MI 48824, USA; Email: Pestka@msu.edu; Tel.: +1-517-353-1709; Fax: +1-517-353-8963; 2 Department of Food Science and Human Nutrition, Michigan State University, East Lansing, MI 48824, USA; 3 Center for Integrative Toxicology, Michigan State University, East Lansing, MI 48824, USA

**Keywords:** deoxynivalenol (DON), translation inhibition, macrophage, monocyte, cytokine, ribosome, ER stress

## Abstract

The trichothecene mycotoxin deoxynivalenol (DON) is commonly encountered in human cereal foods throughout the world as a result of infestation of grains in the field and in storage by the fungus *Fusarium*. Significant questions remain regarding the risks posed to humans from acute and chronic DON ingestion, and how to manage these risks without imperiling access to nutritionally important food commodities. Modulation of the innate immune system appears particularly critical to DON’s toxic effects. Specifically, DON induces activation of mitogen-activated protein kinases (MAPKs) in macrophages and monocytes, which mediate robust induction of proinflammatory gene expression—effects that can be recapitulated in intact animals. The initiating mechanisms for DON-induced ribotoxic stress response appear to involve the (1) activation of constitutive protein kinases on the damaged ribosome and (2) autophagy of the chaperone GRP78 with consequent activation of the ER stress response. Pathological sequelae resulting from chronic low dose exposure include anorexia, impaired weight gain, growth hormone dysregulation and aberrant IgA production whereas acute high dose exposure evokes gastroenteritis, emesis and a shock-like syndrome. Taken together, the capacity of DON to evoke ribotoxic stress in mononuclear phagocytes contributes significantly to its acute and chronic toxic effects *in vivo*. It is anticipated that these investigations will enable the identification of robust biomarkers of effect that will be applicable to epidemiological studies of the human health effects of this common mycotoxin.

## 1. Introduction

Consumption of bread made from overwintered cereal grains infested with the mold *Fusarium* during World War II in the USSR resulted in massive outbreaks of alimentary toxic aleukia (ATA) [1]. ATA, a frequently fatal disease, involves both the immune and gastrointestinal systems and, as its name implies, evokes symptoms that included diarrhea, vomiting, leukopenia, hemorrhage and shock. Human gastroenteritis with nausea, diarrhea and vomiting as primary symptoms has been similarly linked to *Fusarium*-contaminated foods in Japan, Korea [2], China [3] and India [4]. Retrospective studies have demonstrated that a commonality among the fusaria isolated from such outbreaks is their capacity to produce a class of highly toxic secondary metabolites known as trichothecenes.

Trichothecene mycotoxins are low molecular weight (≈200–500 D) sesquiterpenoids that contain both a common 9, 10 double bond and 12, 13 epoxide group as well as varied substituent groups that contribute significantly to their toxic potential. Of the over 200 members of this intriguing family that have been so far described [5,6,7], deoxynivalenol (DON) is very commonly encountered in cereal-based foods throughout the world [8]. High dose, acute exposure of sensitive animal species to DON, most notably pigs (e.g., 15–20 ppm in diet), elicits abdominal distress, increased salivation, malaise, diarrhea and emesis [9,10,11,12,13,14]. Contrastingly, prolonged low dose feeding of DON to experimental pigs and mice (e.g., 1–10 ppm) impairs weight gain, causes anorexia and possibly interferes with nutritional efficiency [13]. DON can stimulate or suppress immune function depending on dose, exposure frequency, timing and the functional immune assay being used [15]. As will be discussed below, this diverse spectrum of effects is likely to result from differences in intensity and duration of kinase signaling and the extent of resultant gene expression.

Large-scale epidemics of *Fusarium* infection in wheat, barley and corn with corresponding DON contamination are increasingly being observed in the U.S. and other parts of the world, possibly as inadvertent outcomes of expanded use of “no-till farming”, inappropriate crop rotation and climate change. Recent biomarker studies indicate that most persons who consume wheat-containing foods are regularly exposed to DON [16,17]. Not surprisingly, there is growing global concern over the possibility of adverse human health outcomes resulting from acute and chronic DON consumption. Understanding the mode of action of natural toxins such as DON facilitates accurate prediction of potential toxic effects, thereby enabling more precise science-based risk assessment and risk management.

Studies in our laboratory and others have revealed that the innate immune system plays an intricate role in DON toxicity. The purpose of this review is to discuss (1) the molecular mechanisms that underlie DON toxicity with a specific focus on mononuclear phagocytes and (2) the relationship between DON-induced proinflammatory gene expression and downstream pathologic sequelae.

## 2. DON Targets Mononuclear Phagocytes

### 2.1. In vitro effects of DON

Primary cultures and cell lines derived from bone marrow, gut epithelium, liver, lymphoid tissue, kidney, lung and various cancers have been employed to assess DON’s toxic effects. Leukocytes, most notably those of mononuclear phagocyte lineage, appear to be particularly responsive to DON [15]. Exposure of macrophages and monocytes to DON at low or moderate concentrations (*i.e.*, partially inhibit translation) will selectively induce proinflammatory gene expression, but extended exposures to high concentrations (*i.e.*, completely inhibit translation) can cause cell death. Susceptibility to apoptosis induction can vary greatly among cell types, with primary cells sometimes being more resistant [18].

### 2.2. Mechanisms for DON inhibition of translation

It has been proposed that trichothecenes including DON inhibit protein synthesis by binding to the peptidyl transferase region of the ribosome and interfering with initiation and elongation [19,20]. In addition to this canonical mechanism, DON might suppress protein synthesis in at least three other ways. For example, incubation of cloned macrophages with DON can result in lesions in the 28s rRNA which might render the 60s subunit non-functional [21]. In addition, DON induces activation of a ribosome-associated kinase known as double-stranded RNA-associated protein kinase (PKR) [22]. Upon activation, PKR can phosphorylate eukaryotic initiation factor 2α (EIF2α), thereby preventing translation [23]. Finally, it has recently been observed that DON can upregulate a large number of microRNAs (miRNAs) associated with selective gene downregulation [24]. Since many of these DON-induced miRNAs specifically correspond to ribosomal proteins, it is possible that those miRNA downregulate ribosome synthesis thus enabling DON-exposed cells to economize and redistribute resources needed for survival.

### 2.3. Mechanisms for DON-induced proinflammatory gene upregulation

Robust upregulation of specific cytokines, chemokines and other inflammation-related proteins by DON *in vitro* is preceded by elevations of their corresponding mRNAs [15] (Table 1). For example, DON induces mRNAs for TNF-α and IL-6 in macrophages [25,26], IL-8 in monocytes [27,28] and IL-2 expression in T cells [29,30]. The premise that DON and other translational inhibitors selectively drive overexpression of specific proteins appears at first to be intuitively contradictory. However, it is not unreasonable to predict that the rapid (e.g., 1 to 2 h) and marked (e.g., 10- to 1000-fold) upregulation of selected mRNAs in cells with partially suppressed translation would dramatically skew the expressed proteome. This concept is supported by comparable observations made for the ribotoxic proteins ricin and Shiga toxin [31,32,33].

Upregulation of mRNA expression by DON involves both transcriptional and post-transcriptional processes [15,34,35,36]. DON induces transcription factor expression (e.g., c-Fos, Fra-2, c-Jun , JunB, EGR1, ATF3) [37,38] and transcription factor activation (e.g., NF-κB, CREB, AP-1 and C/EBP) [29,39,40,41,42]. These transcription factors specifically regulate expression of inflammation- and immune-related genes. DON-induced transactivation has been confirmed using promoter-reporter studies assays [28,34,43,44].

**Table 1:** Genes upregulated by deoxynivalenol in mice.

Gene Family	Gene
Proinflammatory Cytokines	IL-1α, IL-1β, IL-6, IL-11, TNF-α, TGF-β
T Cell Cytokines	IFN-γ, IL-2
Chemokines	MIP-2, MCP-1, Crg-2, CINC-1, MCP-3
Transcription Factors	cFos, cJun, Fra-2, Jun-B, NR4a1
Phosphatases	MKP1, CNAb, Ptpn8, Ptprj
Suppressors of Cytokine Signaling (SOCS)	CIS1,SOCS1, SOCS2 ,SOCS3
Other	Cox-2, C3aR
See references [26,34,48,83,100,120].	

DON also stabilizes mRNAs for TNF-α, IL-6 and COX-2 [25,43,45], and IL-8 [39]. Enhanced mRNA stability relates to AUUUA motifs in the 3'-untranslated region (3'-UTR) of mRNAs that target transcripts for rapid degradation. Involvement of these 3’ UTRs has been verified in studies of DON-induced COX-2 [43] and IL-8 mRNAs [39] stabilization. Notably, mRNA stability in the latter investigation was related to translocation of HuR/Elav-like RNA binding protein 1 (ELAVL1) from the nucleus to the cytosol and its association with 3'-untranslated region of the IL-8 transcript.

Transcriptional and post-transcriptional gene upregulation by DON is mediated by mitogen-activated protein kinases (MAPKs) known to be critical for signal transduction in the immune response. Activation of MAPKs by translational inhibitors was initially described in the landmark paper of Iordanov *et al.* [46] who termed it the “ribotoxic stress response”. Three MAPK families are activated by DON in macrophage and monocyte cultures. These include: extracellular signal regulated protein kinase 1 and 2 (ERK1 and 2); (ii) p54 and p46 c-Jun N-terminal kinase 1 and 2 (JNK 1/2) and (iii) p38 [15].

While both ERK and p38 contribute to DON-induced transactivation of TNF-α, IL-6 and COX-2, only p38 is essential for trichothecene-mediated mRNA stabilization [25,34,43]. Analogous results have been observed for IL-8 in U937 human monocytes [47] and primary human mononuclear blood cell cultures [48]. Consistent with *in vitro* studies, DON sequentially induces (1) p38, ERK and JNK phosphorylation, (2) activation of the transcription factors AP-1, C/EBP, CREB and NF-κB binding, and (3) proinflammatory cytokine mRNA expression in lymphoid tissues of the mouse [49].

**Figure 1:**
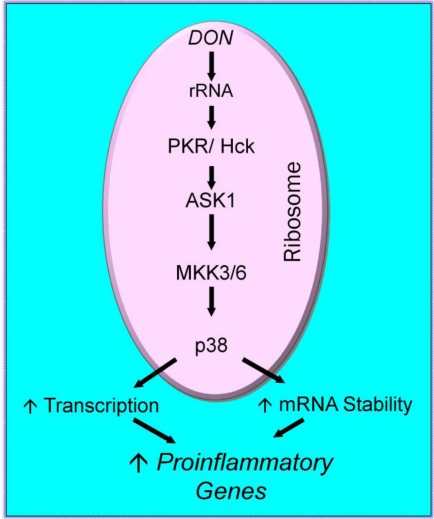
DON-induced ribotoxic stress—Mechanism I. One mechanism for DON-induced ribotoxic stress is proposed to involve: (1) rapid DON uptake and binding to ribosome; (2) activation of ribosomal-associated PKR and Hck; (3) interaction of p38 with the ribosome; (4) p38 phosphorylation and (5) induction of proinflammatory genes.

When specific chemical inhibitors were used to screen for potential upstream kinases that could mediate DON-induced MAPK activation, both PKR and hematopoeitic cell kinase (Hck) were identified as candidate transducers [22,50]. PKR, a widely-expressed serine/threonine protein kinase, causes translational inhibition in an evolutionarily conserved antiviral response by phosphorylating [23]. PKR functions as a signal integrator for ligand-activated stress-activated protein kinase pathways leading to JNK and p38 activation as well as induction of TNF-α, IL-6 and IL-12 expression. Hck, a member of the highly conserved Src-family of cytoplasmic protein tyrosine kinases, is specifically expressed in myelomonocytic cell lineages [51]. Hck transduces extracellular signals that regulate critical cellular processes such as proliferation, differentiation, migration and cytokine upregulation [52]. PKR and Hck are therefore likely to be essential for early steps in the ribotoxic stress response, and, furthermore, mediate initial events resulting in innate immune activation associated with macrophage exposure to DON (Figure 1).

### 2.4. Mechanisms for DON-induced cell death

MAPK activation also precedes trichothecene-mediated apoptosis in RAW 264.7 macrophage and U937 monocyte models suggesting they have an equally important regulatory role in cell death [53]. When the contribution of p38 in mediating apoptosis and survival was investigated in DON-treated RAW 264.7 macrophages [54], it was observed that at concentrations that inhibit translation partially, DON induces p38 and ERK 1/2 phosphorylation within 15 min and this lasts up to 3 h. DON-exposed cells exhibit increased caspase 3-dependent DNA fragmentation within 6 h that is suppressed and potentiated by p38 inhibition and ERK inhibition, respectively. DON evokes BAX translocation to mitochondria and cytochrome C release but does not affect mitochondrial membrane potential. In addition, DON also induces p38-dependent p53 activation. The p53 inhibitor PFTα and p53 siRNA knockdown suppress DON-induced caspase-3 activation and consequent DNA fragmentation. Accordingly, it has been proposed that DON induces competing apoptotic (p38→p53→Bax→Mitochondria→Caspase-3) and survival (ERK→AKT/p90Rsk→Bad) pathways in RAW 264.7 macrophages. Since both PKR and Hck inhibition suppress DON-induced p53 activation, caspase-3 activity and apoptosis, these kinases are also likely to be upstream transducers in MAPK-regulated apoptosis [22,42].

On a cautionary note, while potent trichothecenes such as T-2 toxin cause robust cell death in lymphoid tissues and bone marrow, generally by apoptosis, DON’s effects are more modest [20,53,55,56,57,58,59,60,61]. Exposure of mice to 25 mg/kg DON induces apoptosis in the thymus, bone marrow, spleen and Peyer’s patches [62], whereas exposure to 12.5 mg/kg does not [63,64] suggesting that relatively high DON dose (*i.e.*, 1/2 to 1/3 LD_50_) is required for this programmed cell death. At 100 to 1000 ng/mL, DON induces apoptosis in cloned macrophages and monocytes [22,54,65], T cells [66] and B cells [67]. Treatment with DON of thymus, spleen and bone marrow cultures at 250 to 500 ng/mL for 18 h induces apoptosis [68], but comparatively higher concentrations of DON (1–50 µg/mL) are required to cause modest apoptosis in T-cells, B-cells and IgA+ cells in mouse lymphoid cultures after 8 h [69]. Murine peritoneal macrophages are very resistant to DON even at concentrations up to 5 µg/mL for 12 h [70]. Comparable variability in DON-induced death induction among hematopoietic precursors has been observed [57,71]. Over all, these observations strongly suggest that inherent differences may exist in the balance between pro-apoptotic and anti-apoptotic pathways within individual phenotypes. Extrapolation of apoptosis studies conducted in cloned cells, often derived from tumors, to those carried out in primary cultures or in intact animals must be therefore performed with caution.

## 3. Initiating Events in the Ribotoxic Stress Response

**Figure 2:**
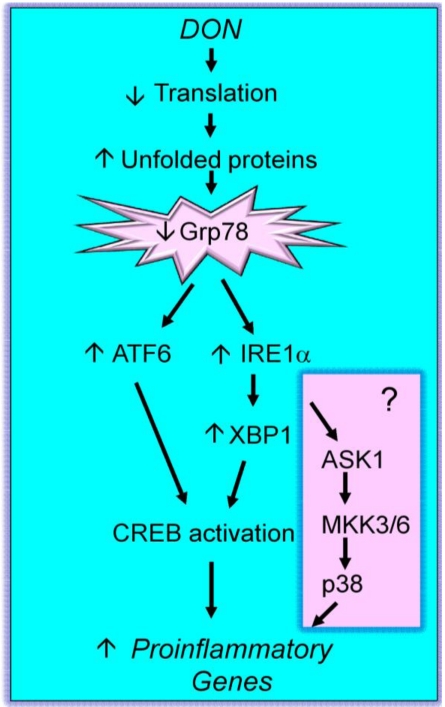
DON-induced ribotoxic stress—Mechanism II. The toxin increases unfolded protein concentration resulting in sequestering and degradation of GRP78 thereby evoking an ER stress. The resultant response includes upregulation of ATF6 and XBP1 resulting in CREB activation. Another hypothetical effect is activation of ASK1-mediated p38 activation (pink shade). Both actions could contribute to proinflammatory gene expression.

Although there is recent evidence that DON interacts with both the 40S and 60S ribosomal units [72], the specific role of the ribosome in DON-induced MAPK activation and proinflammatory gene expression has not been fully delineated. Two possible contributing mechanisms identified to date are the direct activation of ribosome-associated kinases (Figure 1) and indirect activation via endoplasmic reticulum (ER) stress response (Figure 2).

It has been increasingly recognized that cells are able to sense damage-associated molecular patterns and evoke stress responses as a result [73]. We hypothesized that following DON-mediated perturbation or damage to rRNA, the ribosome acts as the initial staging site for MAPKs [74]. DON-treated U937 human monocytes and RAW 264.7 murine macrophages ribosomes were therefore subjected to sucrose density gradient fractionation and fractions immunoblotted for p38. Both total and phosphorylated p38 were found to increase in those fractions containing ribosomal subunits and monosomes. DON induced a similar segregation of JNK and ERK into the ribosomal fractions with concurrent phosphorylation. These data suggest that ribosome might serve as a scaffold in the ribotoxic stress response. It has now been demonstrated that PKR and Hck associate constitutively with ribosomal fractions where they potentially integrate the initial “sensing” of DON-induced ribosomal RNA damage and subsequently mediate intracellular kinase signaling [75] (Figure 1).

DON and other trichothecenes differ from ribosome-inactivating proteins (RIPs) such as ricin because, being small molecules, they do not possess the inherent enzyme capacity to promote 28S rRNA cleavage at the alpha-sarcin/ricin (S/R)-loop (A4256) under cell-free conditions [76]. However, incubation of RAW 264.7 cells with either DON or ricin promotes the cleavage of 28S rRNA at two other sites (A3560 and A4045) in the peptidyl transferase center. Furthermore, both DON and ricin induce RNase activity and RNase L mRNA expression.

Thus, DON can promote intracellular 28S rRNA cleavage, potentially by facilitating the action of endogenous RNases and/or by upregulating RNase expression. Although it remains to be resolved whether these cleavages are upstream or downstream to the actual ribotoxic stress response, the findings support the possibility that DON interacts with the peptidyl transferase region of the 28S rRNA in a similar fashion to ricin. It is tempting to speculate that DON-induced perturbation or damage at this site initiates kinase activation and recruitment. PKR is an attractive candidate for the sensing role since it has a dsRNA binding site that could bind to damaged rRNA.

DON interaction with the ribosome also results in an ER stress response (Figure 2). In support of this contention, DON-treated macrophages markedly decrease expression of cytoplasmic glucose regulated protein (GRP) 78, a chaperone known to mediate ER stress in peritoneal macrophages [18]. Since GRP78 mRNA is unchanged following DON treatment, GRP78 loss likely results from degradation rather than reduced expression. The ubiquitin-proteasome and autophagy-lysosomal pathways constitute two major avenues for protein degradation [77]. DON-induced GRP78 degradation is cathepsin- and calpain-dependent but not proteosome-dependent suggesting an autophagy pathway is involved [18]. Potential mechanisms for DON-induced GRP78 autophagy are not known. Ribosome disruption and/or translation arrest might cause accumulation of misfolded proteins that are sequestered by GRP78. Resultant GRP-containing complexes could enter into the autophagy pathway and be degraded. It should be noted that while the autophagy-lysosomal predominates here, DON has been demonstrated to upregulate genes associated with regulation and structure of the proteosome complex [78] as well as proteins of the ubiquitin-proteosome complex [79].

GRP78 regulates two transcription factors, X-box binding protein 1 (XBP1) and activating transcription factor 6 (ATF6). These bind to cAMP-response element (CRE) and drive expression of CRE-dependent genes. DON treatment increases levels of ATF6 as well as IRE1α protein and its modified products spliced XBP1 mRNA and XBP1 protein [18]. ATF6 but not XBP1 knockdown partially inhibits DON-induced IL-6 expression. 

It is also possible that DON-induced ER stress contributes to MAPK activation. Specifically, it is known that (1) IRE 1 activation mediates ASK1 phosphorylation and (2) DON induces ASK1 phosphorylation [75]. Accordingly, the ER stress response could thus be a second complementary pathway by which DON and other trichothecenes affect innate immune function (Figure 2). Three other translation inhibitors, T-2 toxin, Shiga toxin and ricin, also induce GRP78 degradation, further suggesting this pathway could be a common mechanism for ribotoxic stress.

It should be further noted that while ER stress initially triggers evolutionarily conserved signal-transduction events designed to ameliorate unfolded protein accumulation in the ER, if these events are severe or protracted, they can induce apoptosis. In the future, it will be desirable to identify critical events upstream and downstream of GRP degradation as well as ascertain the comparative contributions of this pathway to upregulation of inflammatory genes and apoptosis.

While two possible mechanisms of ribotoxic stress have been proposed here for DON, it does not preclude the existence of alternative mechanisms for this or other agents. For example palytoxin does not inhibit translating ribosomes under cell-free conditions but requires translating ribosomes to transduce signals that activate JNKI [81]. Osman *et al.* [79] observed in a proteomic analysis of EL-4 thymoma cells that DON induced expression of My-binding protein A (MYBBP1A). This latter protein interacts with many ribosomal proteins and can interact with a number of transcription factors [82].

## 4. Pathological Sequelae to DON-Induced Innate Immune Activation

**Figure 3:**
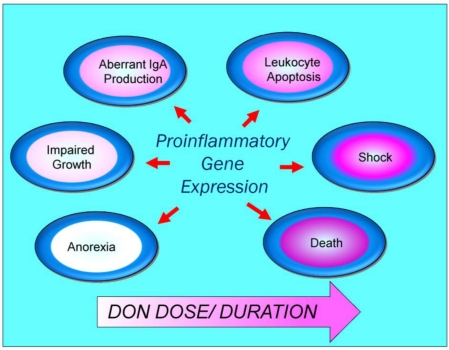
Potential downstream pathological sequelae associated with DON-induced ribotoxic stress.

What is the *in vivo* relevance of the DON-induced proinflammatory gene expression? DON is rapidly distributed throughout the body following oral exposure of mice [83,84] and thus would be present in many tissues containing mononuclear phagocytes. Consistent with *in vitro* studies, DON concomitantly induces a wide array of proinflammatory cytokines and chemokines that are detectable in spleen, liver, kidney and lung [37,49,84,85,86,87,88,89]. Ribotoxic stress in mononuclear phagocytes, with consequent induction of proinflammatory gene expression, is likely to be critical for the induction of acute and chronic sequelae associated with DON poisoning in experimental animals (Figure 3).

Aberrant elevation of inflammatory mediators, often referred to as a cytokine storm [90], mediate the shock-like effects of lipopolysaccharide (LPS) [91] and might likewise contribute to acute toxic effects of DON. Indeed, LPS and other toll-like receptor (TLR) agonists potentiate DON toxicity in mice [62,89,92,93,94,95]. Interestingly, DON has been reported to damage the integrity of intestinal cells and allow increased bacterial translocation [96,97,98,99]. Such collateral damage could greatly magnify DON toxicity.

Proinflammatory cytokines induce several suppressors of cytokine signaling (SOCS). DON upregulates mRNA expression of four well-characterized SOCS (CIS [cytokine-inducible SH2 domain protein], SOCS1, SOCS2, and SOCS3) following cytokine upregulation [100]. DON specifically induces SOCS3 mRNAs in muscle, spleen and liver, in addition to CIS1, SOCS1, and SOCS2 in other tissues. Notably, hepatic SOCS3 mRNA and protein are sensitive indicators of DON exposure. SOCS induction could be an essential feedback mechanism for downscaling proinflammatory gene effects and their subsequent pathological sequelae. Another complementary feedback mechanism reported for DON is the upregulation of mitogen-activated protein kinase phosphatase 1 [86].

Induction of proinflammatory cytokines could contribute DON impairment of appetite and weight gain observed by either directly affecting the brain and nervous system [101] and/or SOCS-mediated deregulation of growth hormone (GH) signaling [102]. In support of the latter, subchronic dietary exposure of young mice to DON decreases hepatic insulin-like growth factor acid labile subunit (IGFALS) mRNA expression and downregulates plasma insulin-like growth factor 1 (IGF1) and IGFALS levels, as well as attenuates weight gain [103]. Liver IGFALS mRNA levels decrease within 2 h of oral exposure to DON (0.5 to12.5 mg/kg body weight), while 0.1 mg/kg body weight DON is without effect. The latter effects co-occur with robust hepatic SOCS3 upregulation with and without exogenous GH treatment. Oral DON exposure in the mouse therefore appears to dysregulate the GH axis by impairing two critical growth-related proteins, IGFALS and IGF1. While their specific contributions to DON-induced food intake and growth retardation still remain to be elucidated, both proteins might be useful as serum biomarkers for DON effect [104].

Prolonged DON feeding to mice causes dramatic elevations in total serum IgA and serum IgA-immune complexes (IgA-IC) and polymeric IgA [105,101]. DON-exposed mice further exhibit kidney mesangial IgA accumulation, electron dense mesangial deposits and hematuria [106]—all hallmarks of human IgA nephropathy, the most common type of glomerulonephritis worldwide [107]. Following removal of DON from mouse diet, toxin-induced elevations in serum IgA, IgA-IC, mesangial IgA and hematuria persist for several months [108]. Peyer’s patches (PP) from DON-exposed mice contain increased numbers of membrane IgA-bearing cells [109]. PP lymphocytes from PP and spleens of DON-fed mice produce significantly more IgA than control cultures prepared from mice fed clean diet [110]. Accordingly, in mice exposed to DON, there is rapid polyclonal activation of IgA-secreting plasma cells in the gut at the PP level and this is similarly reflected in the systemic compartment.

DON does not exert adjuvant effects when orally administered with exogenous mucosal antigens, but rather, polyclonally induces production of IgAs that are reactive with a variety of intestinal and self antigens [111,112,113,114]. Polyspecific, autoreactive IgA may contribute to kidney immune complex deposition or direct binding to the kidney mesangium. IL-6, which is robustly induced upon DON exposure *in vivo* and *ex vivo* [87,115] is known to drive differentiation of B cells to IgA production [116]. The macrophages appear to contribute to IgA production and IgA nephropathy in DON-exposed mice by upregulating IL-6 [117]. Serum IgA, IgA immune complexes, kidney mesangial IgA and hematuria were significantly higher in DON-fed wild-type mice than toxin-fed IL-6 KO [118] suggesting that IL-6 is a requisite cytokine for DON-induced IgA production and resultant IgAN.

Taken together, DON-induced proinflammatory cytokine production by mononuclear phagocytes *in vivo* is likely to contribute to many pathological sequelae associated with exposure to trichothecene.

## 5. Conclusions

Substantive questions remain regarding the risks posed to humans from acute and chronic DON ingestion and how to manage these risks without threatening food security. As demonstrated here, DON dramatically induces MAPK activation *in vitro* and *in vivo* in macrophages and monocytes which in turn, mediate robust induction of both proinflammatory gene expression and, at very high doses, apoptosis. Mechanisms for the DON-induced ribotoxic stress response appear to involve the (1) activation of constitutive ribosomal kinases and mobilization of MAPKs to the damaged ribosome (Figure 1) and (2) autophagy of GRP78 with consequent action of an ER stress response (Figure 2). Downstream pathophysiologic sequelae of DON-induced ribotoxic stress in mononuclear phagocytes include chronic toxic effects such as anorexia, growth and aberrant IgA production as well as acute toxicity at high doses. It is anticipated that current and future investigations will identify robust biomarkers of effect that when coupled with biomarkers of exposure [119] will facilitate epidemiological studies of potential DON-associated human illnesses.
